# Hereditary renal amyloidosis with a variant lysozyme p.Trp82Arg in a Chinese family: case report and literature review

**DOI:** 10.1186/s12882-019-1496-6

**Published:** 2019-08-08

**Authors:** Zhenyu Li, Hui Xu, Dan Liu, Danyang Li, Gang Liu, Su-xia Wang

**Affiliations:** 1 Laboratory of Electron Microscopy, Pathological Center, Peking University First Hospital, Beijing, 100034 People’s Republic of China; 2 Renal Division, Department of Medicine, Peking University First Hospital; Renal Pathological Center, Institute of Nephrology, Peking University; Key Laboratory of Renal Disease, Ministry of Health of China; Key Laboratory of CKD Prevention and Treatment, Ministry of Education of China, Beijing, 100034 People’s Republic of China; 3 Proteomics Laboratory, Medical and Healthy Analytical Center, Peking University Health Science Center, Beijing, 100191 People’s Republic of China

**Keywords:** Hereditary systemic amyloidosis, Lysozyme amyloidosis, P.Trp82Arg, Chinese, Renal involvement

## Abstract

**Background:**

Lysozyme amyloidosis is a rare hereditary systemic amyloidosis with amyloid deposits in various tissues leading to progressive organ failure. It has been mainly reported in developed countries since 1993. Here we report a lysozyme amyloidosis family with variant lysozyme p.Trp82Arg in a Chinese family.

**Case presentation:**

The main clinical manifestation of this case was dominant kidney involvement presenting with proteinuria and decreased renal function. Biopsy of the kidney showed massive amyloid deposits in the glomerular mesangium and subendothelium. Immunohistochemistry and mass spectrometry of renal tissue confirmed the lysozyme nature of the amyloid. DNA sequencing of the peripheral blood leukocytes revealed a single base-pair transition from T to C (TGG/ CGG) of codon 82, leading to the replacement of tryptophan by arginine in the mature protein (p.Trp82Arg). The affected patients in this family also presented with dominant kidney involvement, one of them has been confirmed by IHC and mass spectrometry on his renal biopsy and gene testing as well. As there is no radical therapy for lysozyme amyloidosis, patients were given symptomatic treatment such as antihypertensive drugs and antibiotics. To our knowledge, this is the first report of lysozyme amyloidosis in a Chinese family.

**Conclusions:**

Hereditary amyloidosis with a variant lysozyme of p.Trp82Arg presented with dominant kidney involvement was firstly reported in a Chinese family.

## Background

Amyloidosis encompasses a wide spectrum of protein misfolding disorders, resulting in extracellular insoluble fibrilliar amyloid deposits in various tissues leading to progressive organ failure. The amyloid deposits may be localized or systemic, acquired or hereditary. Systemic amyloidosis can be acquired disorders resulting from immunoglobulin light chain in plasma cell dyscrasias and amyloid A protein in chronic inflammatory conditions, or can be inherited disorders related to gene mutations in the coding regions of transthyretin, apolipoprotein A-I, apolipoprotein A-II, apolipoprotein C-III, apolipoprotein C-II, fibrinogen Aα chain, gelsolin, cystatin C and lysozyme. [1–3]. Acquired monoclonal immunoglobulin light-chain (AL) amyloidosis is the most common form of systemic amyloidosis, while hereditary amyloidosis is thought to be extremely rare [4].

Lysozyme amyloidosis is a hereditary systemic amyloidosis, with autosomal dominant pattern of inheritance. Manifestations of lysozyme amyloidosis are variable, including heartburn, gastric pain, gastrointestinal bleeding, hepatic rupture, renal failure, sicca syndrome and rupture of lymph nodes. Cardiac involvement, peripheral neuropathy and bronchial amyloid deposits seem to be less common than the symptoms mentioned above. Here we report a lysozyme amyloidosis family with dominant kidney involvement, and carried a variant lysozyme p.Trp82Arg, which was identified by immunohistochemistry, mass spectrometry and DNA sequencing. To our knowledge, this is the first report of lysozyme amyloidosis with the p.Trp82Arg variant in a Chinese family.

## Case presentation

**Fig. 1:**
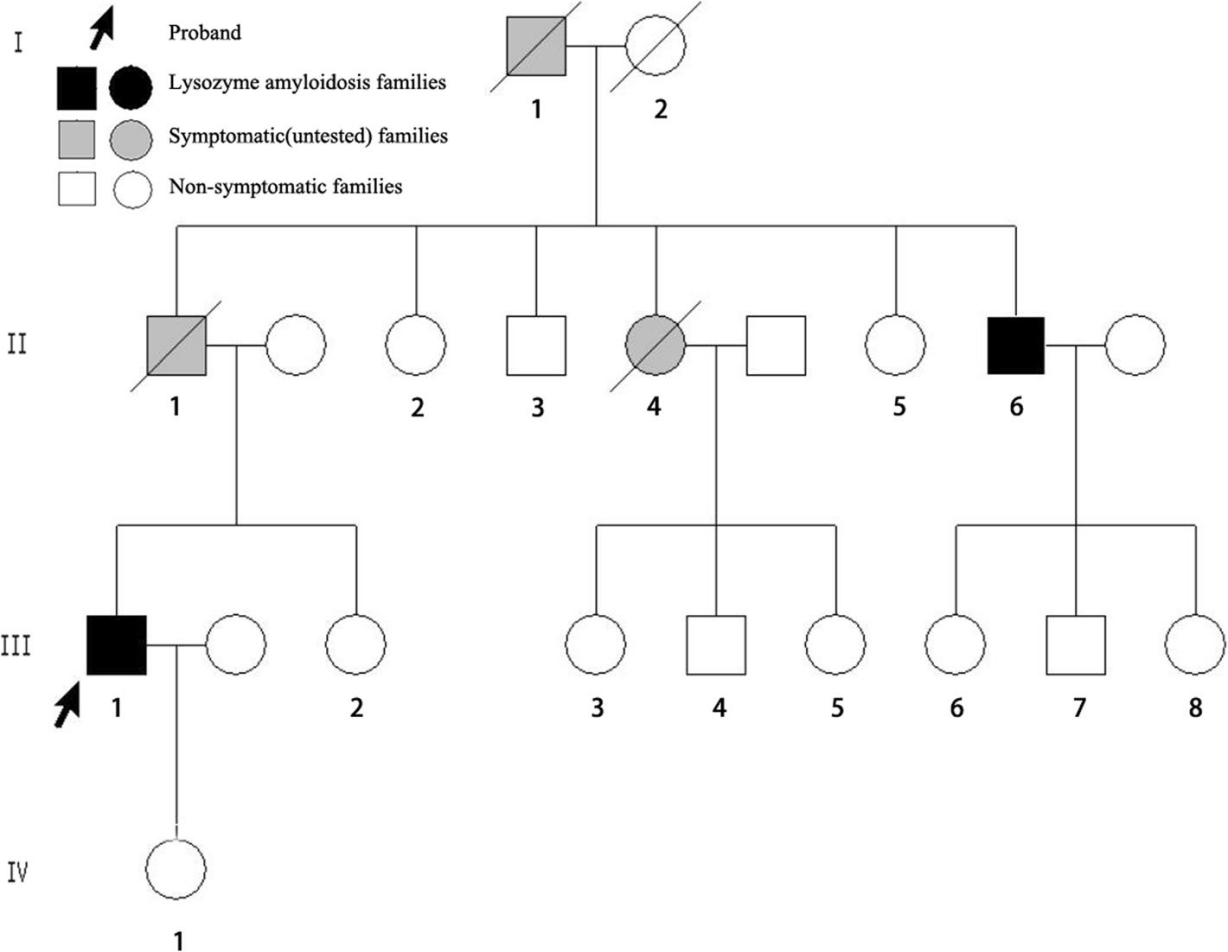
Family tree of the proband’s paternal pedigree. Black symbols denote individuals with the lysozyme p.Trp82Arg mutation while gray symbols denote symptomatic, but untested families. The arrow denotes the proband

A 42-year-old Chinese man with 1-year history of hypertension (the highest pressure was 160/100 mmHg) was admitted into a local hospital for renal dysfunction in June 2016. He had a pain in right foot sole and the first metatarsophalangeal joint for one week. Past medical history includes sleep apnea hypopnea syndrome for five years, which was treated by continuous positive airway pressure. He denied tobacco use and cardiac disease history. The patient was from a family of Chinese ancestry (Fig. 1. III-1). His father (Fig. 1. II-1) and grandfather (Fig. 1. I-1) both died of uremia. His mother had no symptoms of renal or other organs. His father had five siblings, two of whom were reported to have renal disease. Physical exam of the patient was unremarkable, without skin petechiae, macroglossia, hepatosplenomegaly, or peripheral neuropathy which are common signs of amyloidosis. He did not have peripheral edema or other signs of volume overload. Laboratory values demonstrated mild anemia with a hemoglobin concentration of 122 g/L. The sedimentation rate was 68 mm/h and the C-reactive protein level was 8.09 mg/ L. His renal function was impaired with an increased serum creatinine of 172 μmol/L and decreased eGFR of 41.33 mL/min/1.73 m^2^ (estimate by CKD-EPI equation in adults). He had a mild proteinuria with the urine protein of 530 mg/24 h. Immunofixation electrophoresis of serum and urine were both negative for monoclonal immunoglobulin. Liver function and myocardial enzyme examination were normal. CT scan of the lung, ultrasound of the heart and abdominal organs were normal. One month later, he was transferred to Peking University First Hospital in July 2016 for further diagnosis.

**Fig. 2:**
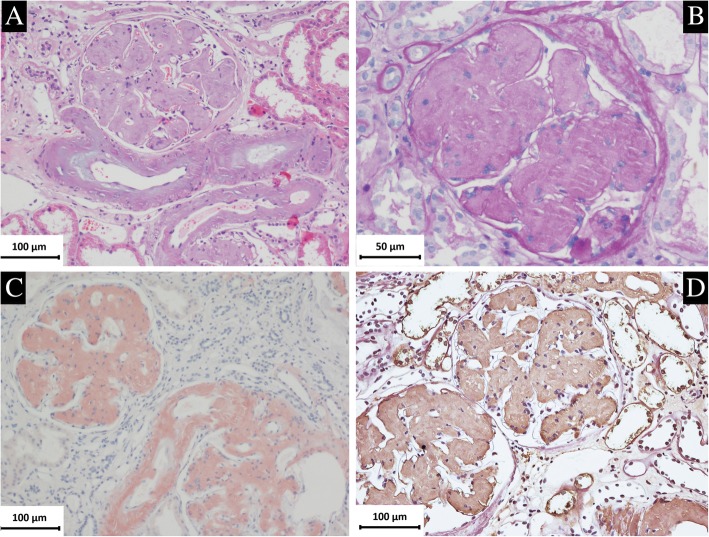
Renal biopsy findings of the proband. **a** Massive homogeneous and lightly stained deposits were found in glomeruli and arteriolar wall (PAS × 200). **b** The amyloid deposits showed PAS positive staining in glomerular mesangium and subendothelium (PAS × 400). **c** Positive Congo red staining in glomerulus and vascular wall (Congo red × 200). **d** IHC revealed positive staining for lysozyme in the glomerular and vascular amyloid deposits(× 200)

Renal biopsy was performed and a strip of renal cortex containing 36 glomeruli were seen under light microscope. Extensive homogeneous and PAS positive stained material was present in glomerular mesangium and subendothelium, also deposited in arteriolar walls [Fig. 2]. These deposits produced apple green birefringence when stained with Congo red and viewed under polarized light. Focal tubular atrophy with interstitial fibrosis and mild infiltration of lymphocytes and monocytes were seen. Routine immunofluorescence showed negative staining for immunoglobulins, complements and light chains (κ and λ).

IHC of the specimen of the patient was strong positive in the renal glomeruli and vessels for lysozyme [Fig. 2]. IHC with antibodies against λ-light chain, κ-light chain, AA amyloid, fibrinogen, transthyretin and gelsolin, apolipoprotein A-I and LECT2 were negative.

**Fig. 3:**
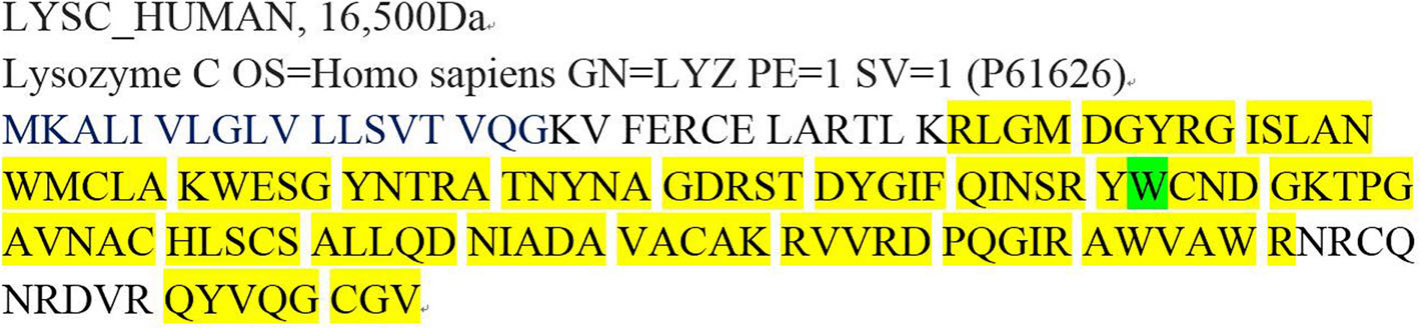
Mass spectrometry-based proteomic analysis of the proband. Yellow part represents covered amino acids, green part at residue 82 represents the replaced amino acid of the wild-type lysozyme

Mass spectrometry-based proteomic analysis confirms the deposits of the patient were lysozyme [Fig. 3].

**Fig. 4:**
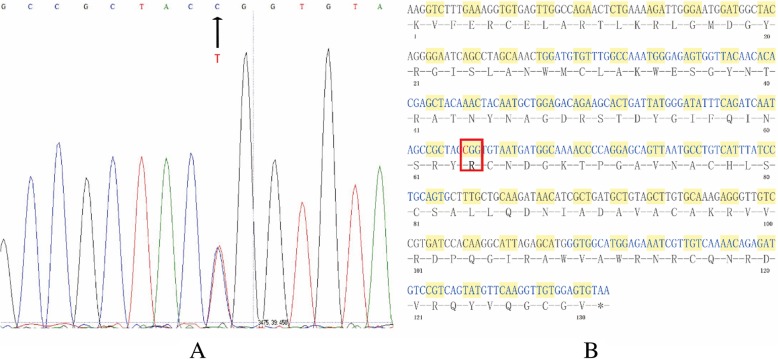
Lysozyme genetic analysis of the proband. **a** Sequence analysis of lysozyme exon 2 showed the presence of both T and C at the first position of codon 82. **b** cDNA sequence and amino acid sequence were showed; red box indicated the exact position of this mutation

Genetic analysis of the lysozyme gene of the patient revealed a single base transversion from T to C at the first position of codon 82 (TGG/ CGG) of exon 2 (Fig. 4. a), leading to the replacement of tryptophan by arginine in the mature protein (p.Trp82Arg, Fig. 4. b). No other base transversions were identified in exon 1 to 4 of lysozyme gene.

The patient was diagnosed as lysozyme amyloidosis. As there is no radical method for this disease, the patient was just given symptomatic treatment such as RAAS blockade, β blocker and CCB to lower blood pressure. After discharge, the patient was in relatively stable condition and took antihypertensive drugs regularly. His serum creatinine was 201 μmol/L and the the urine protein was 3.99 g/24 h in April 2019.

One of the patient’s uncles (Fig. 1. II-6), was admitted into another local hospital for cough, expectoration, haemoptysis, pharyngalgia, dyspnea and a fever (the highest temperature was 38.5 °C) in April 2016. He presented with chronic obstructive pulmonary disease (COPD) for 10 years, renal dysfunction for 7 years and edema of both lower limbs for 2 years. He was a smoker for forty years. He had a urine protein of 12.5 g/24 h with increased serum creatinine of 146 μmol/L. Serum albumin was 24.2 g/L. Urine Bence-Jones protein was negative. Immunofixation electrophoresis of serum and urine was both negative for monoclonal immunoglobulin. Liver function was normal. BNP was 7754Pg/ml. An echocardiogram showed a thicker heart wall of the left ventricular and the ejection fraction was 53%. Chest CT scan revealed emphysema, inflammation of both lower lungs, pleural effusion and mild pericardial effusion. He visited the outpatient of Peking University First Hospital in August 2016 for further diagnosis. His renal biopsy revealed large amyloid deposits in glomeruli and arteriolar walls, which was similar to the proband’s renal biopsy findings. His IHC (pathology pictures were not shown) also showed similar results to the proband’s. Mass spectrometry-based proteomic analysis confirms the deposits were lysozyme, too. He also carried the p.Trp82Arg lysozyme variant. He was treated with antibiotics and oxygen therapy, his fever was gone and the respiratory symptoms relieved.

## Discussion and conclusions

Lysozyme is a bacteriolytic enzyme synthesized by macrophages, hepatocytes and gastrointestinal cells, which was found in biological fluids such as tears, saliva, serum or urine [4]. Lysozyme amyloidosis is an autosomal dominant hereditary systemic amyloidosis which was first described in 1993 by Pepys et al. [5]. Lysozyme variants result in partly folded intermediates, increase the propensity of the protein to deposit in extracellular space under particular circumstances, and therefore progressively disrupt the tissue structures. It is so rare that only about thirty families have been detailed throughout the world. Age at diagnosis can be less than ten years old or more than seventy years old. Eight mutations in the lysozyme gene have been found, and a non-amyloidogenic polymorphism (p.Thr88Asn) has also been reported [6]. The reported lysozyme gene mutations and main clinical manifestations are summarized in Table 1.

**Table 1:** Gene mutations and clinical features of lysozyme amyloidosis

Number	Gene Mutation	Exon	Discovery	Ethnic Background	Clinical Features	Representative Reference
1	p.Ile74Thr	Exon 2	1993	English Indian	Nephropathy, purpura, sicca syndrome, GI* symptoms	[5, 7]
2	p.Asp85His	Exon 2	1993	English	Nephropathy, GI* symptoms, liver rupture, sicca syndrome	[5]
3–1	p.Trp82Arg (T > C)	Exon 2	2002	French	Nephropathy, intestinal obstruction, sicca syndrome	[8]
3–2	p.Trp82Arg (T > A)	Exon 2	2002	Italian, French	GI* symptoms, lymph node rupture, liver rupture	[9]
4	p.Phe75Ile	Exon 2	2003	Italian	Nephropathy	[10]
5	p.Trp130Arg/p.Thr88Asn	Exon 4/Exon 2	2006	German, Mixed Italian, Slovak and German	Nephropathy, GI* symptoms, sicca syndrome, heart failure	[11]
6	p.Asp85Gly	Exon 2	2008	Romanian	Nephropathy, sicca syndrome	[12]
7	p.Tyr72Asn	Exon 2	2012	Swedish	GI* symptoms, sicca syndrome	[13]
8	p.Leu102Ser	Exon 3	2017	American	Nephropathy, GI* symptoms, peripheral neuropathy, possible heart involvement	[14]

*GI represents gastrointestinal

Lysozyme amyloidosis has a large spectrum of clinical manifestations, includes digestive tract (gastrointestinal haemorrhage, abdominal pain, diarrhea, heartburn, nausea, vomiting, maldigestion, weight loss, intestinal perforation), liver and spleen (hepatosplenomegaly, spontaneous rupture of liver or spleen), kidneys (proteinuria, renal dysfunction), lymph nodes (lymphadenopathy, spontaneous rupture of lymph nodes), skin (petechiae, purpura), muscles (amyloid-associated myopathy) [15], lachrymal and salivary glands (sicca syndrome), and very recently cardiac involvement, peripheral neuropathy and bronchial amyloid deposits [16]. Sicca syndrome is often the first symptom, which can precede the diagnosis by several years [17]. Lysozyme amyloid deposits have been found in gastrointestinal tract, liver, spleen, adrenal glands, muscles, vessels, lymph nodes, bone marrow, gallbladder [18] and pancreatic tissue [4] in affected patients. It seems that there is no significant correlation between the mutation pattern and the clinical presentations. Although there are different manifestations between families, even between those with the same mutation, the clinical manifestations are very similar among affected individuals in the same family [19]. Therefore, it is important for physicians to investigate family history of the patients carefully. Given that AL amyloidosis has a relatively fast natural history and chemotherapy can be used in AL amyloidosis, it is critical for physicians to make the correct diagnosis. Many methods can be used if necessary such as immunohistochemical staining, mass spectrometry and genetic sequencing as we have described above, and scintigraphic imaging for serum amyloid P can also be used (mainly performed in Europe now) [20]. There is no radical therapy for lysozyme amyloidosis, treatment is based on supportive and symptomatic therapy. Liver or kidney transplant may be useful as a palliative method for patients with spontaneous liver rupture or renal failure. Although some patients may relapse as lysozyme can be produced again, organ transplant is justifiable for young patients as it can prolong their lives [15].

As for our report, the proband and his uncle were confirmed to have p.Trp82Arg mutation, which was first described in a French family in 2002 by Valleix et al. [8]. Until now, only about 9 families with this mutation have been detailed in the literature (This is the tenth case), most of them are Italian or French origin. To our knowledge, this is the first report of p.Trp82Arg variant in a Chinese family. In this family, DNA sequence analysis reveals a heterozygous single base-pair transition from T to C (TGG/ CGG) of codon 82 of lysozyme exon 2 in the proband and his uncle, leading to the replacement of tryptophan by arginine in the mature protein. The most noticeable clinical presentation was renal impairment in this family, and affected individuals do not have gastrointestinal manifestations due to amyloid deposition. Valleix et al. reported a family with renal failure, small bowel infarction and sicca syndrome caused by p.Trp82Arg (T > C) [8]. Besides, five families with T to A substitution also lead to the same amino acid change (p.Trp82Arg) [4] [16] [18] [9, 21], but manifestations of these families are mainly digestive symptoms. Only one of the five family [9] has a family member who had alterations of the renal function test and mild proteinuria (0.6 g/24 h), a renal biopsy was performed but no evidence of amyloidosis was seen in the kidney of the patient. Nucleotide substitution type of the other three families remains unclear. It seems that there is some kind of correlation between the two different nucleotide substitutions (T to C substitution and T to A substitution) and the manifestations, but it still needs further study. Renal progression varies between patients. In a retrospective evaluation of lysozyme amyloidosis patients in the UK National Amyloidosis Centre, five of seven amyloidotic renal dysfunction patients reached ESRD. Median time from discovery of renal dysfunction to ESRD was 11.0 years [19]. Kidney transplant may be useful for patients with end-stage renal failure. In addition, this mutation (p.Trp82Arg) may have other manifestations such as sicca syndrome, rupture of abdominal lymph nodes and recurrent pulmonary infections. It is notable that our patients also have respiratory symptoms such as sleep apnea hypopnea syndrome (the proband) and COPD (the proband’s uncle). COPD of the uncle is probably related to his tobacco consumption. As for apnea hypopnea syndrome of the proband, he didn’t have central nervous system disease, BMI of him was 24.22 kg/m^2^. Benyamine et al. reported a case in which the patient carrying the p.Trp82Arg lysozyme variant had amyloid deposits in the trancheobronchial leading to recurrent pneumonia. We infer that the proband may also have amyloid deposits in his airways, make his airways narrow and lead to sleep apnea hypopnea syndrome. But the proband didn’t undergo endoscopy, we are unable to confirm the exact cause.

In conclusion, we report a lysozyme amyloidosis family presented with dominant kidney involvement, and associated with variant lysozyme p.Trp82Arg, which was identified by immunohistochemistry, mass spectrometry and DNA sequencing. To our knowledge, this is the first report of lysozyme amyloidosis in a Chinese family, and physicians should be aware that Asians may also suffer from this disease.

## Data Availability

All data generated or analyzed in this study are included in this article.

## Abbreviations

AL: Immunoglobulin light-chain amyloidosis
CCB: Calcium channel blockerCOPD: chronic obstructive pulmonary disease
ESRD: End-stage renal disease
GI: Gastrointestinal
IHC: Immunohistochemistry
RASS: Renin-angiotensin-aldosterone system
